# Association of long working hours with psychological distress in men with pregnant partners: An observational study from the Japan Environment and Children’s Study

**DOI:** 10.1371/journal.pone.0326864

**Published:** 2025-06-25

**Authors:** Hidekuni Inadera, Kenta Matsumura, Haruka Kasamatsu, Junko Sakai, Akiko Tsuchida, the Japan Environment and Children’s Study (JECS) Group

**Affiliations:** 1 Department of Public Health, Faculty of Medicine, University of Toyama, Toyama City, Japan; 2 Toyama Regional Center for JECS, University of Toyama, Toyama City, Japan; 3 Graduate School of Health Sciences, Aomori University of Health and Welfare, Aomori City, Japan; Tokyo Medical and Dental University: Tokyo Ika Shika Daigaku, JAPAN

## Abstract

**Background:**

It has been suggested that working long hours affects workers’ mental health, although findings have been inconsistent. In this study, we investigated the association of working hours with psychological distress in a population of Japanese men with pregnant partners, using data from the Japan Environment and Children’s Study.

**Methods:**

Data from 44,996 men were analyzed and weekly working hours were classified into six groups. The Kessler Psychological Distress Scale (K6) was used to assess mental health. Each of the six items were assessed on a 5-point scale (0–4), with a total score of 0–24 and higher scores indicating greater psychological distress. A total score of 5–12 was considered to indicate moderate psychological distress and a score of ≥13 to indicate severe psychological distress. To investigate the association of working hours with psychological distress, multinomial logistic regression analysis was performed to calculate odds ratios (ORs) and 95% confidence intervals (CIs).

**Results:**

The results showed that after adjusting for covariates, weekly working hours was positively associated with moderate and severe psychological distress. Compared with men who worked ≤40 h per week, those who worked >55 to ≤65 h or >65 h per week had significantly higher ORs (95% CIs) for moderate psychological distress, 1.12 (1.03–1.21) and 1.34 (1.24–1.45), respectively, and those working >65 h per week had significantly higher OR, 1.84 (1.47–2.32) for severe psychological distress. For these two outcomes, a significant p for trend (<.0001) was observed in both the crude and adjusted models.

**Conclusion:**

The results of this study suggest that the greater time constraints resulting from working long hours are associated with psychological distress in Japanese men with pregnant partners.

## Introduction

Working long hours is associated with negative effects on health that reduce worker productivity and increase health care expenditures [1]. In Japanese, the term *karoshi* refers to sudden death due to overworking [2]. *Karoshi* is often caused by coronary heart disease or stroke and may be due to repeated triggering of the stress response [3]. Previous studies have reported an increased risk of coronary heart disease and stroke among individuals working ≥55 h per week compared with those working 35–40 h per week [1–3].

Working long hours is also thought to be associated with mental illness, including depression. Previous studies have shown a negative mental effect of working long hours [4–9]. However, other studies have indicated that working long hours may not be associated with psychological distress or depression [10–12]. One study reported that men who worked overtime more frequently showed a 40% decrease in sub-clinical depression compared with men who worked overtime less frequently [13]. Four recent systematic reviews also reported limited evidence of the association between working long hours and depression [14–17]. This discrepancy in the results may be related to the study populations. Stronger associations were reported in studies based on occupational cohorts in a single organization or a specific occupation compared with those derived from the general working population [14]. Therefore, the effect of working long hours on psychological distress in a large nationwide cohort remains to be clarified.

Previous studies indicated that depression in men during pregnancy and the postpartum period is nearly twice the rate of depression in the adult male population overall [18,19]. Perinatal psychological distress in men with pregnant partners can affect the child’s aggressive behavior [20] and child maltreatment [21] after childbirth. Thus, psychological distress in expectant fathers is important for family health. Among Organization for Economic Co-operation and Development (OECD) countries, Japan is well known for its long working hours [22,23]. In western countries, the range of working hours for men is reported to be narrower compared with Japan [12,24]. Given that overtime work is prevalent in Japan, additional empirical evidence would accurately demonstrate the association between long working hours and mental health in men with pregnant partners.

The objective of this study was to examine the association of working long hours with psychological distress in a large population of Japanese men with pregnant partners. We sought to analyze the potential exposure–response relations between working hours and psychological distress in men with pregnant partners, using the data of the Japan Environment and Children’s Study (JECS).

## Materials and methods

### Study design

JECS is the largest birth cohort study in Japan. The primary objective of the JECS is to assess the effects of environmental factors on the health and development of children. Participants were recruited between January 2011 and March 2014, and a total of 103,057 pregnancies were registered at 15 regional centers throughout Japan. The rationale and study design of JECS were previously reported by Kawamoto *et al*. [25]. The research coordinator introduced and explained JECS to pregnant women (mother participants) undergoing antenatal checkups and obtained their consent. Their male partners (father participants) were recruited after the mothers had agreed to participate in the study. These expectant fathers are the subject of the present study. Consent was obtained from these fathers when they accompanied their partners to antenatal checkups or when they came to visit while their partners and infants were in the hospital after delivery. The number of fathers enrolled was about half that of the mothers [26].

All procedures that contributed to the present work were carried out in accordance with the Declaration of Helsinki as revised in 2008. All procedures involving human subjects in the JECS were reviewed and approved by the Ministry of the Environment’s Institutional Review Board on Epidemiological Studies (100910001) and the ethics committees of all participating institutions. All participants in this study provided written informed consent. The research protocol of the present study was approved by the Institutional Review Board of the University of Toyama (R2023177).

### Study data

**Fig 1:**
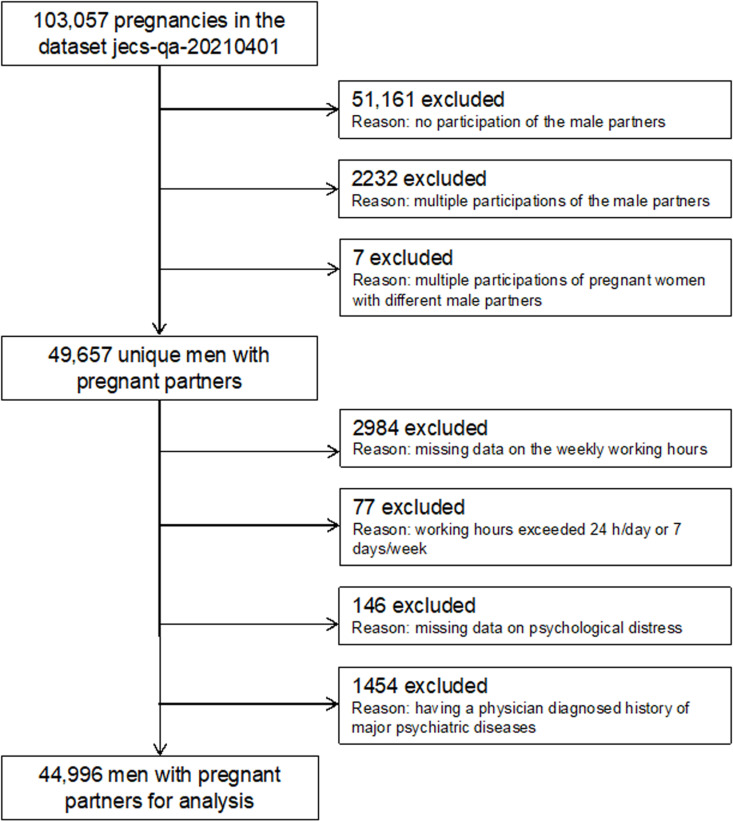
Participant flow diagram. Flow diagram of the recruitment and exclusion process for men with pregnant partners in this study.

The dataset used in this study, jecs-qa-20210401 (jecs-ta-20190930), was initially published in October 2019 and finalized in February 2022. The data were accessed for research purposes on April 23rd, 2024. The dataset contains records for 103,057 pregnancies. Records were excluded as follows: 51,161 related to the male partners not participating in the study, 2,232 related to partners who participated multiple times, and 7 related to the pregnant women participating with different male partners. This left registered responses from 49,657 unique men with pregnant partners. We subsequently excluded 2,984 records due to obviously incorrect answers regarding the men’s weekly working hours, 77 of which indicated that the men worked >24 h a day or >7 days a week, as well as 146 records with missing answers regarding psychological distress. Participants who had a history of physician-diagnosed major psychiatric diseases were also excluded to avoid reverse causality (1,454) because these individuals may have been engaged in jobs with shorter working hours as a result of their mental health. Finally, data from 44,996 men with pregnant partners were analyzed (Fig 1).

### Measurements

Information on demographic characteristics, medical history, physical and mental health status, lifestyle, occupation, and socioeconomic status was collected using a self-administered questionnaire, which was distributed to the male participants during the period between their partner’s pregnancy and when their infants turned 1 month old.

### Exposure

The number of hours worked per week was calculated by multiplying the typical number of hours worked per day, including overtime, by the number of workdays per week obtained from the self-administered questionnaire. We asked the following two questions: “How many hours do you work per day?” and “How many days do you work per week?” The working hours are considered to be those at the time of recruitment. Thus far, studies in Western countries have tended to categorize long working hours into three analytical categories of 41–48, 49–54, and ≥55 h/week, comparing these with standard working hours of 35–40 h/week [1]. In the present study, we divided the working hours into six categories, which may allow for a more significant conclusion and yield results that can be utilized identify dose–response relationships. We grouped the participants into six categories by working hours as follows: ≥ 0 to ≤40 h, > 40 to ≤45 h, > 45 to ≤50 h, > 50 to ≤55 h, > 55 to ≤65 h, and >65 h per week, as in previous studies [27,28].

### Outcomes

The Japanese version of the Kessler Psychological Distress Scale (K6), a six-item self-reported questionnaire developed to detect psychological distress in the general population [29], was used to assess mental health. The K6 consists of six questions that examine the frequency during the last 30 days of the following items: (1) nervousness, (2) hopelessness, (3) restlessness or fidgetiness, (4) being so depressed that nothing could cheer you up, (5) feeling that everything is an effort, and (6) worthlessness. The six items are assessed on a 5-point scale (0–4), with a total score of 0–24 and higher scores indicating greater psychological distress. The validity of the Japanese version of the K6 has been previously established, and two cutoff points have been proposed: 4/5, which is psychometrically the most appropriate, and 12/13, which is the most widely used [30,31]. A score of 5–12 was considered to indicate moderate psychological distress and a score of ≥13 severe psychological distress [30–33].

### Covariates

A series of variables on known or suspected risk factors affecting work hours and psychological distress were considered as covariates in the association analysis. Model 1 was adjusted for age (<25, 25 to <30, 30 to <35, 35 to <40, ≥ 40 years) and socioeconomic status, namely, educational attainment (≤12, 12 to <16, ≥ 16 years) and annual household income (<4, 4 to <6, ≥ 6 million yen). In model 2, we added the following as possible confounders: body mass index (<18.5, 18.5 to <25, ≥ 25 kg/m^2^), marital status (married, single), smoking status (never, former, current), alcohol intake (never, former, current), physician-diagnosed history of any physical disease (yes, no), autistic trait defined as an Autism-Spectrum Quotient [34] score of ≥7 (yes, no), and job category (managers; professionals and technicians; clerical support workers; sales workers; service workers; protective service workers; skilled agricultural, forestry, and fisheries workers; craft and manufacturing workers; drivers and machine operators; construction or mining workers; package deliverers, cleaners, hand packers, and other elementary workers; full-time homemakers, students, or unemployed; occupations not classified above).

### Statistical analysis

To estimate the risk of two levels of psychological distress (i.e., moderate and severe) according to the six categories of weekly working hours, we performed a multinomial logistic regression analysis and calculated the odds ratios (ORs) and 95% confidence intervals (CIs). Odds ratios (95% CIs) were calculated for the ordinal cases of moderate and severe psychological distress, with a K6 score of ≤4 and working ≤40 h per week groups set as the reference. Two-sided *p* values of <0.05 were taken as statistically significant. In tests for trend, we assigned the numbers 1–6 to the six weekly working hours categories and evaluated them as a continuous variable. Missing data were treated by using multiple imputation. We created 10 imputed datasets by using chained equations, and the results were combined using Rubin’s rules. Data were analyzed using SAS ver. 9.4 (SAS Institute Inc., Cary, NC).

## Results

The participant characteristics are shown in Table 1. Around 60% of the participants reported working ≤50 h per week, 9.6% worked 50 to ≤55 h, 16.3% worked 55 to ≤65 h, and 15.3% worked >65 h. The most common highest educational attainment was junior high school or high school (41.5%). About 70% of the participants were in their 30s or older and roughly the same percentage had household incomes of <6 million yen.

**Table 1:** Characteristics of the participants analyzed in the study (N = 44,996, all male).

Variable	Weekly working hours					
	≤40	>40 to ≤45	>45 to ≤50	>50 to ≤55	>55 to ≤65	>65
Age, years						
<25	800 (8.3)	340 (5.8)	731 (6.7)	321 (7.4)	443 (6.0)	463 (6.7)
25 to <30	2369 (24.7)	1422 (24.2)	2568 (23.5)	1009 (23.3)	1693 (23.1)	1631 (23.6)
30 to <35	2967 (30.9)	1935 (32.9)	3666 (33.5)	1430 (33.0)	2488 (33.9)	2293 (33.2)
35 to <40	2277 (23.7)	1463 (24.9)	2622 (24.0)	1063 (24.6)	1817 (24.8)	1662 (24.1)
≥40	1165 (12.1)	705 (12.0)	1328 (12.1)	497 (11.5)	874 (11.9)	824 (11.9)
Missing	24 (0.3)	19 (0.3)	26 (0.2)	8 (0.2)	27 (0.4)	26 (0.4)
Body mass index, kg/m^2^						
<18.5	346 (3.6)	245 (4.2)	361 (3.3)	153 (3.5)	261 (3.6)	231 (3.4)
18.5 to <25	6613 (68.9)	4120 (70.0)	7498 (68.5)	3027 (69.9)	5036 (68.6)	4626 (67.1)
≥25	2565 (26.7)	1471 (25.0)	2983 (27.3)	1106 (25.6)	1971 (26.9)	1965 (28.5)
Missing	78 (0.8)	48 (0.8)	99 (0.9)	42 (1.0)	74 (1.0)	77 (1.1)
Marital status						
Married	9229 (96.1)	5751 (97.7)	10563 (96.6)	4178 (96.5)	7078 (96.4)	6634 (96.2)
Single	306 (3.2)	114 (1.9)	305 (2.8)	123 (2.8)	231 (3.2)	212 (3.1)
Missing	67 (0.7)	19 (0.3)	73 (0.7)	27 (0.6)	33 (0.5)	53 (0.8)
Highest education level, years						
≤12	4230 (44.1)	2071 (35.2)	4745 (43.4)	1836 (42.4)	2953 (40.2)	2816 (40.8)
12 to <16	2298 (23.9)	1500 (25.5)	2409 (22.0)	977 (22.6)	1670 (22.8)	1643 (23.8)
≥16	2906 (30.3)	2231 (37.9)	3618 (33.1)	1444 (33.4)	2599 (35.4)	2319 (33.6)
Missing	168 (1.8)	82 (1.4)	169 (1.5)	71 (1.6)	120 (1.6)	121 (1.8)
Annual household income, million JPY						
<4	3544 (36.9)	1834 (31.2)	3920 (35.8)	1681 (38.8)	2700 (36.8)	2536 (36.8)
4 to <6	3027 (31.5)	2031 (34.5)	3415 (31.2)	1293 (29.9)	2333 (31.8)	2088 (30.3)
≥6	2322 (24.2)	1646 (28.0)	2806 (25.7)	1039 (24.0)	1843 (25.1)	1767 (25.6)
Missing	709 (7.4)	373 (6.3)	800 (7.3)	315 (7.3)	466 (6.4)	508 (7.4)
Smoking status						
Never	3027 (31.5)	2030 (34.5)	2974 (27.2)	1209 (27.9)	1840 (25.1)	1776 (25.7)
Former	2759 (28.7)	1676 (28.5)	3035 (27.7)	1230 (28.4)	2038 (27.8)	1845 (26.7)
Current	3664 (38.2)	2098 (35.7)	4775 (43.6)	1804 (41.7)	3334 (45.4)	3162 (45.8)
Missing	152 (1.6)	80 (1.4)	157 (1.4)	85 (2.0)	130 (1.8)	116 (1.7)
Alcohol intake						
Never	2177 (22.7)	1271 (21.6)	2202 (20.1)	903 (20.9)	1524 (20.8)	1416 (20.5)
Former	364 (3.8)	175 (3.0)	373 (3.4)	146 (3.4)	281 (3.8)	252 (3.7)
Current	7037 (73.3)	4420 (75.1)	8334 (76.2)	3266 (75.5)	5511 (75.1)	5201 (75.4)
Missing	24 (0.3)	18 (0.3)	32 (0.3)	13 (0.3)	26 (0.4)	30 (0.4)
History of any physical diseases						
No	3106 (32.4)	1675 (28.5)	3723 (34.0)	1453 (33.6)	2456 (33.5)	2270 (32.9)
Yes	6496 (67.7)	4209 (71.5)	7218 (66.0)	2875 (66.4)	4886 (66.6)	4629 (67.1)
Autistic trait						
No	8943 (93.1)	5523 (93.9)	10187 (93.1)	3995 (92.3)	6864 (93.5)	6426 (93.1)
Yes	638 (6.6)	349 (5.9)	731 (6.7)	321 (7.4)	464 (6.3)	451 (6.5)
Missing	21 (0.2)	12 (0.2)	23 (0.2)	12 (0.3)	14 (0.2)	22 (0.3)
Job category						
Managers	454 (4.7)	229 (3.9)	383 (3.5)	155 (3.6)	279 (3.8)	319 (4.6)
Professionals and technicians	2588 (27.0)	1986 (33.8)	3501 (32.0)	1346 (31.1)	2058 (28.0)	1795 (26.0)
Clerical support workers	1254 (13.1)	777 (13.2)	944 (8.6)	293 (6.8)	493 (6.7)	273 (4.0)
Sales workers	553 (5.8)	530 (9.0)	1007 (9.2)	561 (13.0)	1133 (15.4)	978 (14.2)
Service workers	1031 (10.7)	581 (9.9)	880 (8.0)	401 (9.3)	884 (12.0)	1022 (14.8)
Protective service workers	448 (4.7)	183 (3.1)	424 (3.9)	95 (2.2)	219 (3.0)	457 (6.6)
Skilled agricultural, forestry, and fisheries workers	126 (1.3)	46 (0.8)	213 (2.0)	84 (1.9)	168 (2.3)	155 (2.3)
Craft and manufacturing workers	1800 (18.8)	982 (16.7)	1489 (13.6)	527 (12.2)	699 (9.5)	381 (5.5)
Drivers and machine operators	287 (3.0)	134 (2.3)	285 (2.6)	142 (3.3)	379 (5.2)	587 (8.5)
Construction or mining workers	305 (3.2)	171 (2.9)	1145 (10.5)	454 (10.5)	526 (7.2)	436 (6.3)
Package deliverers, cleaners, hand packers, and other elementary workers	148 (1.5)	58 (1.0)	134 (1.2)	33 (0.8)	116 (1.6)	119 (1.7)
Full-time homemakers, students, or unemployed	134 (1.4)	8 (0.1)	23 (0.2)	11 (0.3)	19 (0.3)	20 (0.3)
Occupations not classified above	237 (2.5)	101 (1.7)	253 (2.3)	113 (2.6)	149 (2.0)	148 (2.2)
Missing	237 (2.5)	98 (1.7)	260 (2.4)	113 (2.6)	220 (3.0)	209 (3.0)

Data are presented as the number (%).

The ORs and 95% CIs for the association of weekly working hours with moderate or severe psychological distress are shown in Table 2. Compared with men who worked ≤40 h per week, those who worked >55 to ≤65 h or >65 h per week had significantly higher ORs for moderate psychological distress in both the crude and adjusted models. Among men who worked >55 h per week, the adjusted ORs (95% CI) for moderate psychological distress ranged from 1.12 (1.03–1.21; > 55 to ≤65 h) to 1.34 (1.24–1.45; > 65 h). Men who worked >65 h per week had significantly higher ORs for severe psychological distress: 1.69 (1.35–2.11) in model 1 and 1.84 (1.47–2.32) in model 2. Trend tests revealed a positive association of weekly working hours with moderate and severe psychological distress in both models (*p* for trend < .0001).

**Table 2:** Cases of psychological distress according to weekly working hours.

	Weekly working hours						
	≤40 (n = 9602)	>40 to ≤45 (n = 5884)	>45 to ≤50 (n = 10941)	>50 to ≤55 (n = 4328)	>55 to ≤65 (n = 7342)	>65 (n = 6899)	*p* value for trend
Moderate psychological distress							
Cases, *n*	1693	1062	2002	814	1398	1486	
Prevalence, %	17.6	18.1	18.3	18.8	19.0	21.5	
Crude model	1.00 (Ref.)	1.02 (0.94, 1.11)	1.05 (0.97, 1.12)	1.08 (0.98, 1.18)	1.10 (1.02, 1.19)	1.30 (1.20, 1.40)	<.0001
Adjusted model 1^a^	1.00 (Ref.)	1.04 (0.96, 1.13)	1.05 (0.98, 1.13)	1.08 (0.98, 1.18)	1.11 (1.03, 1.20)	1.31 (1.21, 1.41)	<.0001
Adjusted model 2^b^	1.00 (Ref.)	1.04 (0.95, 1.13)	1.07 (0.999, 1.16)	1.09 (0.99, 1.20)	1.12 (1.03, 1.21)	1.34 (1.24, 1.45)	<.0001
Severe psychological distress							
Cases, *n*	154	70	167	53	134	173	
Prevalence, %	1.60	1.19	1.53	1.22	1.83	2.51	
Crude model	1.00 (Ref.)	0.74 (0.56, 0.99)	0.96 (0.77, 1.20)	0.77 (0.56, 1.06)	1.16 (0.92, 1.47)	1.66 (1.33, 2.07)	<.0001
Adjusted model 1^a^	1.00 (Ref.)	0.77 (0.58, 1.03)	0.97 (0.78, 1.22)	0.77 (0.56, 1.06)	1.18 (0.94, 1.50)	1.69 (1.35, 2.11)	<.0001
Adjusted model 2^b^	1.00 (Ref.)	0.81 (0.61, 1.09)	1.05 (0.84, 1.32)	0.81 (0.59, 1.12)	1.27 (0.998, 1.61)	1.84 (1.47, 2.32)	<.0001

Values show the imputed data for the 44,996 men with pregnant partners.

^a^ Adjusted for age, education, and annual household income.

^b^ Adjusted for age, BMI, marital status, education, annual household income, smoking status, alcohol intake, physician-diagnosed history of any physical disease, autistic trait, and job category.

## Discussion

The objective of this study was to examine the association of working hours with psychological distress in a large population of Japanese men with pregnant partners. Our results revealed a positive association of weekly working hours with moderate and severe psychological distress. Working >55 h per week may increase the risk of moderate psychological distress compared with working ≤40 h per week. In addition, we observed that working >65 h per week was associated with severe psychological distress. Previous studies have reported that working >55 h per week may have an adverse effect on workers’ mental health [5,8,35], and our results were in line with this threshold. One possible biological mechanism for this association is that longer working hours reduce the time for rest and sleep, resulting first in carryover fatigue followed by a decline in mental health. Moreover, expectant fathers may experience stress associated with the birth of a baby and the responsibilities inherent in becoming a parent, which may exacerbate psychological distress.

In a previous study, we found that working longer hours was associated with lower parenting behavior in men [27], which in turn was associated with higher psychological distress in their partners [32]. The number of hours that men work throughout their partner’s pregnancy may have an impact on their partner as well as their children. Thus, the working hours of expectant fathers are important not only in terms of the psychological distress experienced by the expectant fathers but also the health of the mother and child.

In this study, about 15% of the participants worked >65 h per week. The men in this group showed a greater tendency toward severe psychological distress compared with those working ≤40 h per week. The data analyzed in this study were collected in the early 2010s. The Ministry of Internal Affairs and Communications conducted a survey during the same period and found that 13.7% of men in the entire working population aged ≥15 years worked ≥60 h per week [36]. The participants in this study were working-age expectant fathers, and thus younger compared with the overall working population. The mean age of our participants was 32.7 ± 5.8 years, while that of the overall male workforce in 2011–2014 was about 41.8 years [37]. It is therefore reasonable to assume that the data in our study reflect a general trend in working hours in Japan in the early 2010s.

Since these data were obtained, policies aimed at reducing long working hours in Japan have been implemented by the Japanese government. In 2014, the “Act on the Promotion of Preventive Measures Against Karoshi and Other Overwork-Related Health Disorders” was passed with the aim of developing a national initiative to prevent overwork-related disorders [38]. In 2018, labor reform laws were enacted with the goal of changing the way people work. For example, companies are prohibited from making employees work more than 65 h per week [39]. This restriction is in line with our finding that working 65 h per week had significantly higher ORs for severe psychological distress. According to a 2022 survey conducted by the Ministry of Internal Affairs and Communications, the percentage of men working ≥60 h per week was 8.1%, indicating a downward trend [23,40]. The work environment conditions during the study period might differ from the work environment conditions today. Moreover, the COVID-19 pandemic that began in 2020 led to an increase in remote work, and thus the working conditions and the mental states of workers may have changed as a result. The actual effects of these circumstances on psychological distress need to be further examined in contemporaneous cohorts.

The association between long working hours and psychological distress has been reported to be stronger in Asian countries than in Western countries [17]. This might be due to differences in national and organizational culture. Japan, like South Korea, has a high degree of collectivism and emphasizes group harmony and loyalty to the company, which can lead to overwork without sufficient organizational or social support for workers, thereby intensifying the adverse impact on mental health [41,42]. Thus, the association between working hours and the mental health of workers varies depending on the regional context. Accordingly, the results of the present study may not be generalizable to workers in other countries, especially those in Western countries.

This study had several strengths. The findings can be considered reliable because they were obtained using data collected from a large nationwide cohort [26], allowing us to examine the relationship between two factors among tens of thousands of participants. Moreover, this study was not based on occupational cohorts or workers within specific occupations but instead included populations in 15 regions across Japan. Second, compared with previous studies conducted outside Japan, we were able to analyze a wider range of working hours. We examined the relationship between working hours and psychological distress and found a dose–response relationship. In western countries, the mean prevalence of employees working ≥55 h a week is low at 7.2% (range, 1.2%–16.6%) [1,24]. In Denmark, only 6.5% of the general population worked >48 h per week [12]. The trend toward working long hours in Japan in the early 2010s allows for an examination of the impact of a wide range of working hours on psychological distress.

Several limitations of the study should also be mentioned. Because the study has a cross-sectional and observational design, no conclusions can be drawn about causality. For example, employees with mental health challenges are likely to have lower productivity, which forces them to work longer hours to do the same job as healthy workers. Second, it has been reported that more demanding jobs, lower job control, lower decision latitude, and effort–reward imbalance may increase the risk depression onset [43,44]. However, the present study was unable to consider these aspects of the fathers’ jobs. Third, personality traits, concurrent life stressors, and stress outside work were not considered. Fourth, the study sample comprised expectant fathers and so the results of this study may be difficult to generalize directly to other cohorts. Moreover, the participants in this study were skewed in terms of younger age groups compared with workers overall. Fifth, only about half as many fathers were registered in the JECS compared with mothers. This might have resulted in a selection bias in which fathers whose work prevented them from being present at the time of face-to-face recruitment were not included in the study. Sixth, the data were obtained from self-reported questionnaires rather than objective measures or structured interviews, and thus information bias and measurement errors may be present. There is a possibility that self-reported working hours might trigger socially desirable answers, that is, a tendency to overestimate shorter working hours and underestimate longer working hours compared with using time diaries [45]. Therefore, it is desirable to collect data on absolute overtime by using an objective measure.

## Conclusions

The results of this study suggest that working long hours is positively associated with psychological distress in Japanese men whose partners are pregnant. Working >55 h per week may lead to moderate psychological distress. The results also provide valuable insights for policymakers and healthcare providers working to maintain the mental health of men with pregnant partners. For example, the workplace may play an important role in assessing and alleviating psychological distress in men with pregnant partners by reducing the time spent at work and promoting a better work–life balance. Companies can also ensure that expectant fathers receive social support, which may effectively reduce the negative impact of long working hours [46].

## Abbreviations

CI: confidence interval
JECS: Japan Environment and Children’s Study
OECD: Organization for Economic Co-operation and Development
OR: odds ratio
